# Supplementary material for: The association between bullous pemphigoid and cognitive outcomes in middle-aged and older adults: A systematic review and meta-analysis
Source: PLoS One. 2023 Nov 30;18(11):e0295135. doi: 10.1371/journal.pone.0295135 (PMC10688758; doi:10.1371/journal.pone.0295135)
Supplement: S2 Table — (DOCX) [file pone.0295135.s002.docx]

S2 Table. Search terms included for each library search

| **PUBMED 32**  (((Bullous Pemphigoid[Title/Abstract]) OR (Pemphigoid[Title/Abstract])) OR (Pemphigoids[Title/Abstract])) AND (((((((((((((((Dementia[Mesh]) OR (Dementias[tiab])) OR (Amentia[tiab])) OR (Amentias[tiab])) OR ("Senile Paranoid Dementia"[tiab])) OR ("Dementias, Senile Paranoid"[tiab])) OR ("Paranoid Dementia, Senile"[tiab])) OR ("Paranoid Dementias, Senile"[tiab])) OR ("Senile Paranoid Dementias"[tiab])) OR ("Familial Dementia"[tiab])) OR ("Dementias, Familial"[tiab])) OR (((((((((((((((("Alzheimer Disease"[Mesh]) OR ("Alzheimer Dementia"[tiab])) OR ("Alzheimer Dementias"[tiab])) OR ("Dementia, Alzheimer"[tiab])) OR ("Alzheimers Disease"[tiab])) OR ("Dementia, Senile"[tiab])) OR ("Senile Dementia"[tiab])) OR ("Dementia, Alzheimer Type"[tiab])) OR ("Alzheimer Type Dementia"[tiab])) OR ("Alzheimer-Type Dementia"(ATD[tiab]))) OR ("Alzheimer Syndrome"[tiab])) OR ("Alzheimers Diseases"[tiab])) OR ("Alzheimer Diseases"[tiab])) OR ("Alzheimers Diseases"[tiab])) OR ("Familial Alzheimer Disease"(FAD[tiab]))) OR ("Presenile Alzheimer Dementia"[tiab]))) OR (((((((("Dementia, Vascular"[Mesh]) OR ("Dementias, Vascular"[tiab])) OR ("Vascular Dementias"[tiab])) OR ("Vascular Dementia"[tiab])) OR ("Dementia, Subcortical Vascular"[tiab])) OR ("Arteriosclerotic Dementia"[tiab])) OR ("Binswanger Disease"[tiab])) OR ("Vascular Dementia, Subcortical"[tiab]))) OR (((((((("Mixed Dementias"[Mesh]) OR ("Dementia, Mixed"[tiab])) OR ("Mixed Dementia"[tiab])) OR ("Mixed-Etiology Dementias"[tiab])) OR ("Dementia, Mixed-Etiology"[tiab])) OR ("Mixed Etiology Dementias"[tiab])) OR ("Mixed-Etiology Dementia"[tiab])) OR ("Multiple-Etiology Dementias"[tiab])))) AND ((((((("Cohort Studies"[Mesh]) "Follow-Up Studies"[Mesh]) OR "Longitudinal Studies"[Mesh]) OR "Prospective Studies"[Mesh]) OR "Retrospective Studies"[Mesh]) OR "Cross-Sectional Studies"[Mesh]) OR ((((Cohort[tiab]) OR Cross-Sectional[tiab]) OR "Cross Sectional"[tiab]) OR Longitudinal[tiab]) AND (((elderly[tiab]) OR "old adults"[tiab])) OR ((Aged[Mesh]) OR "Middle Aged"[Mesh]))) |
| --- |
| **EMBASE (Elsevier) 88**  ((('Bullous Pemphigoid':ti,ab) OR (Pemphigoid:ti,ab)) OR (Pemphigoids:ti,ab)) AND (((((((((((((((Dementia/exp) OR (Dementias:ti,ab)) OR (Amentia:ti,ab)) OR (Amentias:ti,ab)) OR ('Senile Paranoid Dementia':ti,ab)) OR ('Dementias, Senile Paranoid':ti,ab)) OR ('Paranoid Dementia, Senile':ti,ab)) OR ('Paranoid Dementias, Senile':ti,ab)) OR ('Senile Paranoid Dementias':ti,ab)) OR ('Familial Dementia':ti,ab)) OR ('Dementias, Familial':ti,ab)) OR (((((((((((((((('Alzheimer Disease'/exp) OR ('Alzheimer Dementia':ti,ab)) OR ('Alzheimer Dementias':ti,ab)) OR ('Dementia, Alzheimer':ti,ab)) OR ('Alzheimers Disease':ti,ab)) OR ('Dementia, Senile':ti,ab)) OR ('Senile Dementia':ti,ab)) OR ('Dementia, Alzheimer Type':ti,ab)) OR ('Alzheimer Type Dementia':ti,ab)) OR ('Alzheimer-Type Dementia' (ATD:ti,ab))) OR ('Alzheimer Syndrome':ti,ab)) OR ('Alzheimers Diseases':ti,ab)) OR ('Alzheimer Diseases':ti,ab)) OR ('Alzheimers Diseases':ti,ab)) OR ('Familial Alzheimer Disease' (FAD:ti,ab))) OR ('Presenile Alzheimer Dementia':ti,ab))) OR (((((((('Dementia, Vascular'/exp) OR ('Dementias, Vascular':ti,ab)) OR ('Vascular Dementias':ti,ab)) OR ('Vascular Dementia':ti,ab)) OR ('Dementia, Subcortical Vascular':ti,ab)) OR ('Arteriosclerotic Dementia':ti,ab)) OR ('Binswanger Disease':ti,ab)) OR ('Vascular Dementia, Subcortical':ti,ab))) OR (((((((('Mixed Dementias'/exp) OR ('Dementia, Mixed':ti,ab)) OR ('Mixed Dementia':ti,ab)) OR ('Mixed-Etiology Dementias':ti,ab)) OR ('Dementia, Mixed-Etiology':ti,ab)) OR ('Mixed Etiology Dementias':ti,ab)) OR ('Mixed-Etiology Dementia':ti,ab)) OR ('Multiple-Etiology Dementias':ti,ab)))) AND ((((((('Cohort Studies'/exp) 'Follow-Up Studies'/exp) OR 'Longitudinal Studies'/exp) OR 'Prospective Studies'/exp) OR 'Retrospective Studies'/exp) OR 'Cross-Sectional Studies'/exp) OR ((((Cohort:ti,ab) OR Cross-Sectional:ti,ab) OR 'Cross Sectional':ti,ab) OR Longitudinal:ti,ab) AND (((elderly:ti,ab) OR 'old adults':ti,ab)) OR ((Aged/exp) OR 'Middle Aged'/exp)) |
| **WEB OF SCIENCE 153**  ((("Bullous Pemphigoid") OR (Pemphigoid)) OR (Pemphigoids)) AND (((((((((((((((Dementia) OR (Dementias)) OR (Amentia)) OR (Amentias)) OR ("Senile Paranoid Dementia")) OR ("Dementias, Senile Paranoid")) OR ("Paranoid Dementia, Senile")) OR ("Paranoid Dementias, Senile")) OR ("Senile Paranoid Dementias")) OR ("Familial Dementia")) OR ("Dementias, Familial")) OR (((((((((((((((("Alzheimer Disease") OR ("Alzheimer Dementia")) OR ("Alzheimer Dementias")) OR ("Dementia, Alzheimer")) OR ("Alzheimers Disease")) OR ("Dementia, Senile")) OR ("Senile Dementia")) OR ("Dementia, Alzheimer Type")) OR ("Alzheimer Type Dementia")) OR ("Alzheimer-Type Dementia"(ATD))) OR ("Alzheimer Syndrome")) OR ("Alzheimers Diseases")) OR ("Alzheimer Diseases")) OR ("Alzheimers Diseases")) OR ("Familial Alzheimer Disease"(FAD))) OR ("Presenile Alzheimer Dementia"))) OR (((((((("Dementia, Vascular") OR ("Dementias, Vascular")) OR ("Vascular Dementias")) OR ("Vascular Dementia")) OR ("Dementia, Subcortical Vascular")) OR ("Arteriosclerotic Dementia")) OR ("Binswanger Disease")) OR ("Vascular Dementia, Subcortical"))) OR (((((((("Mixed Dementias") OR ("Dementia, Mixed")) OR ("Mixed Dementia")) OR ("Mixed-Etiology Dementias")) OR ("Dementia, Mixed-Etiology")) OR ("Mixed Etiology Dementias")) OR ("Mixed-Etiology Dementia")) OR ("Multiple-Etiology Dementias")))) AND ((((((("Cohort Studies") "Follow-Up Studies") OR "Longitudinal Studies") OR "Prospective Studies") OR "Retrospective Studies") OR "Cross-Sectional Studies") OR ((((Cohort) OR Cross-Sectional) OR "Cross Sectional") OR Longitudinal) AND (((elderly) OR "old adults")) OR ((Aged) OR "Middle Aged")) |
